# Case report: Thymoid differentiated carcinoma of thyroid: Two cases

**DOI:** 10.3389/fsurg.2023.1112315

**Published:** 2023-04-27

**Authors:** Yanjie Zhao, Jiafeng Liu

**Affiliations:** Department of Thyroid and Hernia, The First Affiliated Hospital of Gannan Medical College, Ganzhou, China

**Keywords:** thymoid-differentiated carcinoma of thyroid, case report, misdiagnose, distinguish, tracheotomy

## Abstract

**Objective:**

Thymoid carcinoma of the thyroid gland is a rare thyroid tumor, which is often presented in case reports.

**Methods:**

The clinical data of two patients with thymic carcinoma of the thyroid gland were retrospectively reviewed.

**Results:**

Case 1: a middle-aged woman who was admitted to the hospital because of “progressive enlargement of the anterior cervical mass for 8 months.” Color Doppler ultrasound and CT showed malignant tumor with high possibility of bilateral cervical lymph node metastasis. Total thyroidectomy and bilateral central cervical lymph node dissection were performed. Lymph node biopsy showed the metastasis of small cell undifferentiated thyroid carcinoma. Because the biopsy pathological result was not consistent with the pathology of the primary lesion, immunohistochemistry was performed again, and the final diagnosis was thymic carcinoma in the thyroid gland. Case 2: the patient was an elderly man who was admitted to the hospital due to hoarseness for half a month. During the operation, the tumor invaded the trachea, esophagus, internal jugular vein, common carotid artery, and surrounding tissues. Palliative resection of the tumor was performed. The tumor postoperative pathology suggested thymoid carcinoma of the thyroid gland. It recurred and compressed the trachea 4 months after the operation, resulting in dyspnea of the patient, and finally tracheotomy was performed to alleviate the symptoms.

**Conclusion:**

Case 1 showed multiple differences in pathological diagnosis, suggesting that the lack of specific imaging and clinical manifestations of thymoid-differentiated thyroid carcinoma made the diagnosis so difficult. Case 2 progressed rapidly, suggesting that thymoid-differentiated thyroid carcinoma was not always inert, and the treatment and follow-up should follow the principle of individualization.

## 1. Case profile

**Figure 1:**
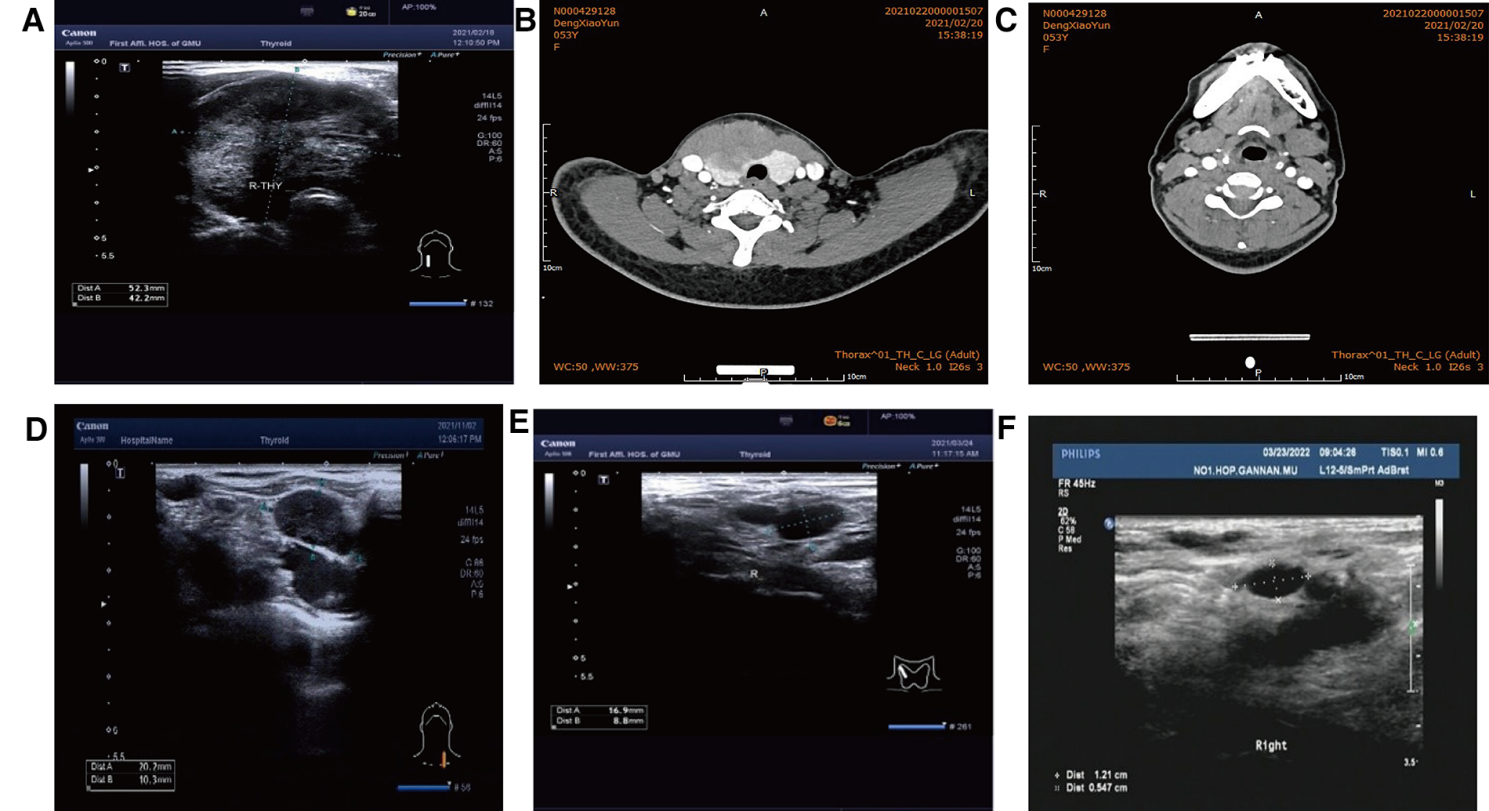
(**A**) Color ultrasound (52 mm × 42 mm hypoecho was seen in the right lobe of the thyroid gland, occupying almost the whole right lobe, with clear boundary and blood flow signals visible inside). (**B**) CT (irregular low-density mass, about 61 mm × 40 mm in size, unclear boundary, moderately uneven enhancement on enhanced scan). (**C**) Postoperative cervical CT. (**D**) Before radiotherapy. (**E**) 1 month after radiotherapy. (**F**) Lymph node shrinkage after local radiotherapy was observed by lymph node color ultrasonography 6 months after radiotherapy.

Case 1: A 53-year-old female patient was presented to our department due to “progressive enlargement of the anterior cervical mass for 8 months.” Color ultrasound indicated the following: hypoechoic right lobe of thyroid, Ti-RADS 4A; no abnormal swollen lymph nodes were found in bilateral neck (Figure 1A). Neck Computed Tomography (Neck CT) showed internal thyroid mass, high possibility of thyroid cancer, and multiple small lymph nodes in bilateral neck regions III and IV and the anterior superior mediastinum (Figure 1B). Inspection result was calcitonin (CT): 0.88 pg/ml and carcinoembryonic antigen (CEA): 7.49 ng/ml. Total thyroidectomy and bilateral central lymph node dissection were performed under general anesthesia, and the postoperative specimens were submitted for examination. During the operation, we could see that the right lobe of the thyroid gland was significantly enlarged, and the tumor was about 50 mm long, hard and without capsule, and the right anterior neck ribbon muscle was observed to be adhesion. The adhesion of the right sternothyroid muscle was also removed during the resection.

**Figure 2:**
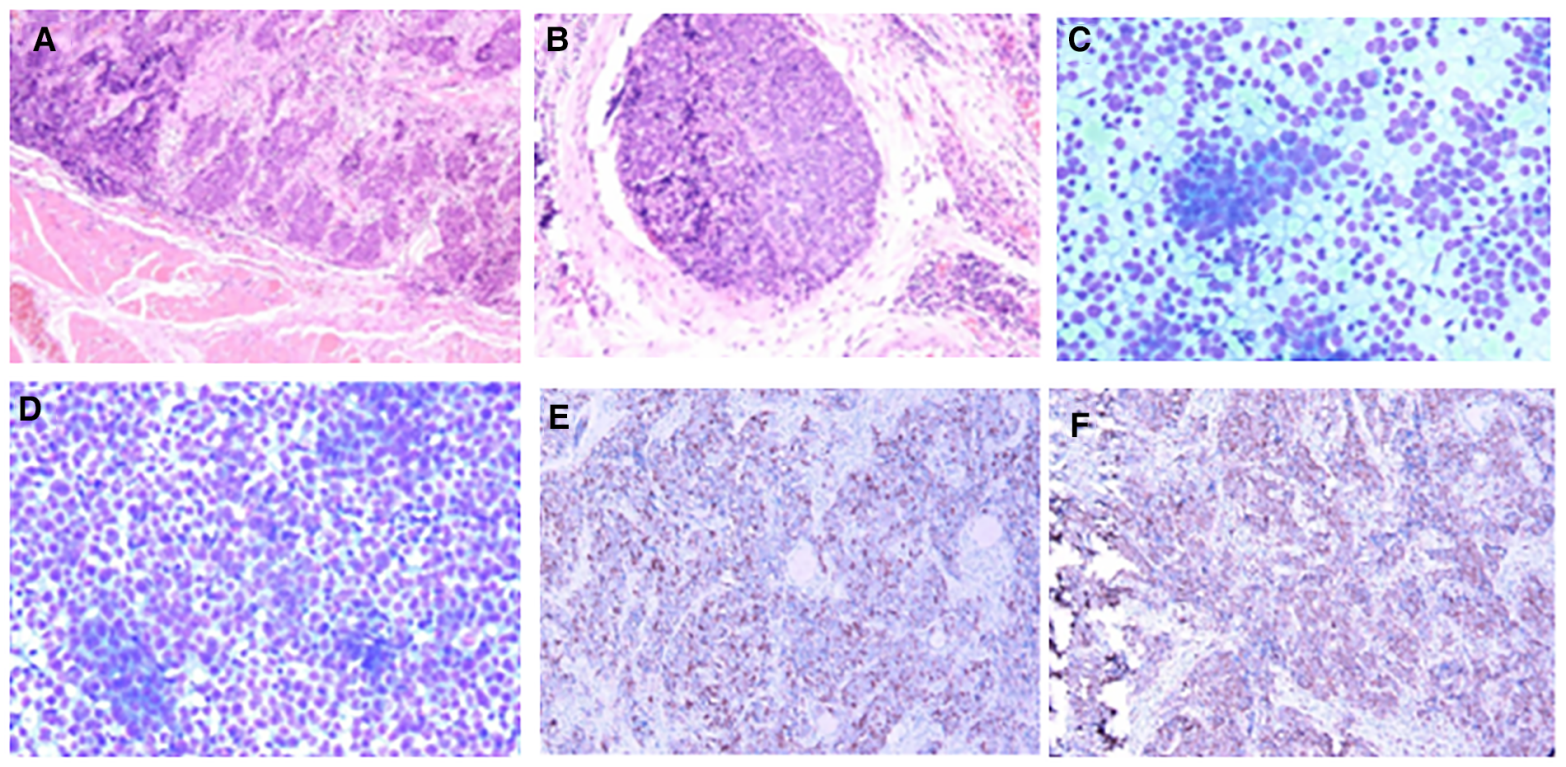
Pathology of case 1: (**A**,**B**) Routine postoperative pathology, inclined to myeloid carcinoma. (**C**,**D**) Lymph node puncture pathology, indicating small cell undifferentiated carcinoma. (**E**,**F**): Immunohistochemistry: CD117 (+) and CD5 (+).

Postoperative pathology: (1) (Thyroid) malignant tumor, which tended to be medullary carcinoma. Immunohistochemical examination was needed to assist the diagnosis. The tumor invaded the capsule and skeletal muscle, and there was tumor thrombus in the vascularization, but no obvious nerve bundle invasion was observed. The peripheral thyroid showed chronic lymphocytic thyroiditis. (2) (Left neck node) no metastasis was observed (0/2). (3) (Right neck lymphatic node) no metastasis was observed (0/6). (4) (Anterior laryngeal lymph node) no metastasis was observed (0/1) (Figures 2A,B). Immunohistochemistry: thyroid was considered as poorly differentiated squamous cell carcinoma. Further clinical examination is recommended to exclude metastatic tumors. Immunohistochemistry: CT (−), Tg (−), CEA (−), CgA (−), Syn (−), TTF-1 (−), CK (+), CK5/6 (+), P53 (+), Ki-67 (45%+), GATA-3 (−), GCDFP-15 (−), CD56 (−), PAX-8 (−), HBME-1 (−), galectin-3 (+), ER (−), and PR (−). Reexamination results at 1 month after operation: (1) Bilateral cervical lymphadenopathy. (2) Thyroid surgery: no obvious spheroid occupying was found in the thyroid area (Figure 1C). Reexamination results at 9 months after operation: (1) Bilateral cervical lymph nodes were enlarged, and metastasis was considered. (2) There was no obvious sphere occupation in the thyroid region. Further biopsy indicated the smear (left neck lateral lymph node biopsy): combined with clinical and cellular morphology, it was consistent with small cell thyroid undifferentiated carcinoma metastasis (Figures 2C,D). Calcitonin (CT) (chemiluminescence method): <0.50 pg/ml; carcinoembryonic antigen (chemiluminescence) 10.3 ng/ml; Neck CT: absence of thyroid after operation, multiple enlarged lymph nodes in bilateral neck region V. Because the lymph node biopsy pathology of the patient was not consistent with the postoperative pathology, the postoperative specimen was resubmitted for immunohistochemistry, and intrathyroid thymic carcinoma (carcinoma with thymoid differentiation) was found. Immunohistochemistry: CT (−), Tg (−), CEA (−), CgA (−), Syn (−), TTF-1 (−), CK (+), CK5/6 (+), P53 (+), Ki-67 (45%+), GATA-3 (−), GCDFP-15 (−), CD56 (−), PAX-8 (−), HBME-1 (−), galectin-3 (+), ER (−), PR (−), P40 (+), P63 (+), CD117 (+), and CD5 (+) (Figures 2E,F). Radiotherapy of cervical lymph nodes was performed. Three months after radiotherapy, the patient returned to the hospital for review, and bilateral cervical lymph nodes in the V region were reduced. Chest and upper abdomen enhanced CT scan showed no clear abnormalities. Color ultrasonography of cervical lymph nodes was performed in June, and several hypoechoic lymph nodes were detected in the III, IV, and VI regions of the left neck. Larger lymph nodes were located in the IV region, the size of which was 10 mm × 6.5 mm, the portal structure was unclear, and blood flow signals were visible. Six months after radiotherapy, several hypoechoic lymph nodes were detected in regions III, IV, and VI of the right neck. The larger lymph nodes were located in region IV, with a size of 12 mm × 5.4 mm. The portal structure was unclear, and blood flow signals could be seen inside (Figure 1F).

**Figure 3:**
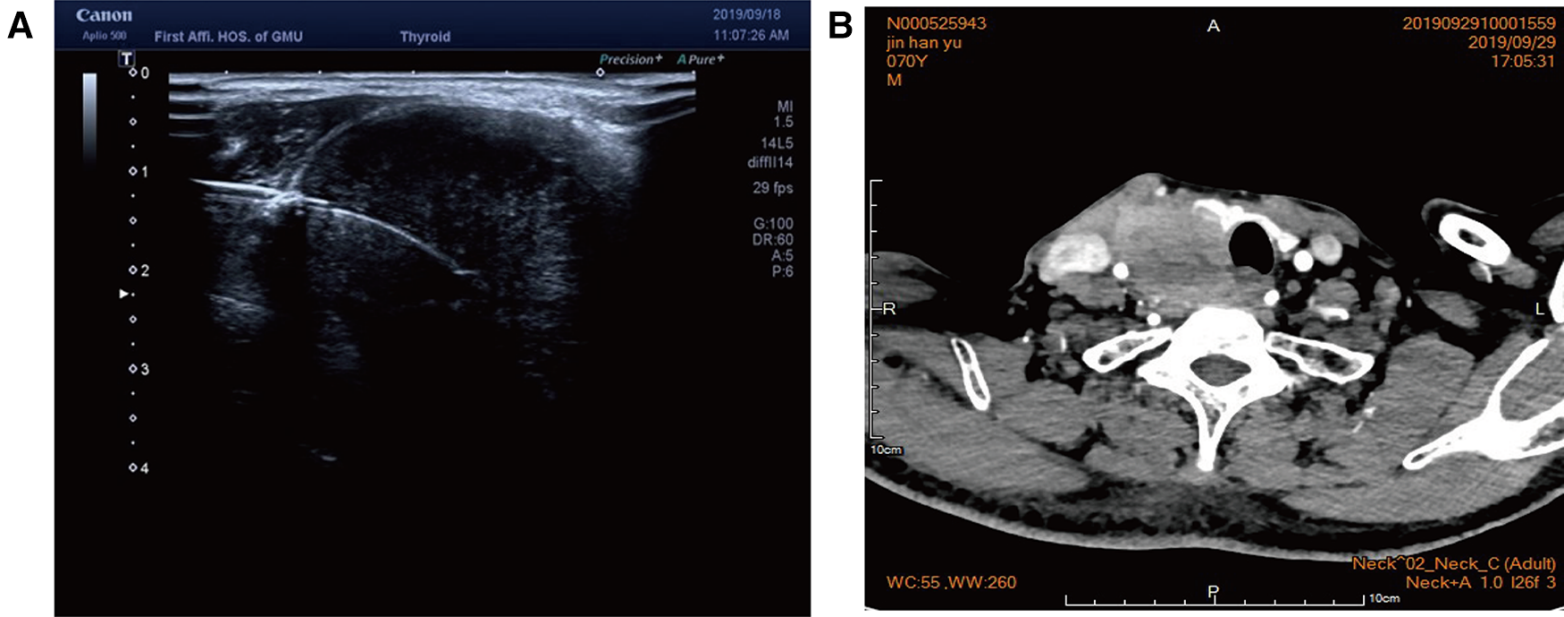
(**A**) Color ultrasound showed hypoecho in the right lobe of the thyroid gland, about 44 mm × 40 mm × 49 mm in size, with unclear boundary. (**B**) Contrast-enhanced CT of the neck showed a mass in the right lobe of the thyroid, with infiltration of the surrounding internal jugular vein, common carotid artery, trachea, and esophagus.

**Figure 4:**
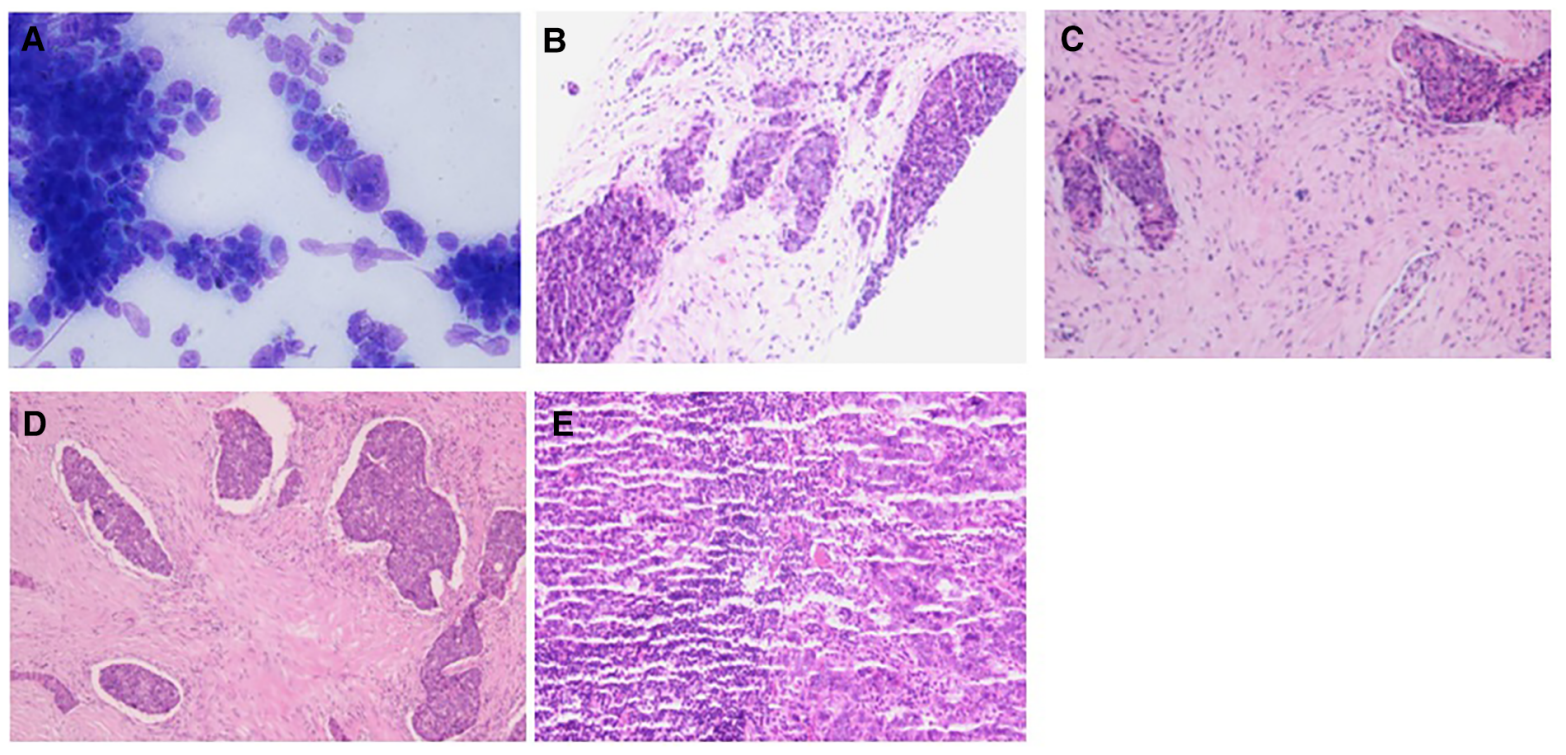
Pathology of case 2: (**A**) The follicular epithelial cells of obvious heterotopy were observed, and it has a papillary structure—fine needle aspiration. (**B**,**C**) A malignant tumor is suggested—coarse needle puncture. (**D**,**E**) Thymoid-differentiated carcinoma is suggested.

Case 2: An elderly male patient was admitted to the hospital due to “hoarseness for half a month.” Color ultrasound showed hypoecho in the right lobe of the thyroid, about 44 mm × 40 mm × 49 mm in size, with unclear boundary (Figure 3A). Plain CT scan indicated the following: (1). Multiple small nodules in both lungs. Follow-up was recommended to exclude metastasis. (2) The right lobe of thyroid was occupied; contrast-enhanced CT of the neck showed a mass in the right lobe of the thyroid, with infiltration of the surrounding internal jugular vein, common carotid artery, trachea, and esophagus (Figure 3B). combined ultrasound or CT enhancement examination showed thyrotropin (chemiluminescence) 9.5 μIU/ml and carcinoembryonic antigen (chemiluminescence) 7.1 ng/ml. Under the guidance of color ultrasound, fine needle aspiration biopsy showed obvious abnormal follicular epithelial cells with papillary structure, which was consistent with malignancy (Figure 4A). Color ultrasound-guided coarse needle biopsy: (right lobe of thyroid puncture) malignant tumor, to be examined by immunohistochemistry to assist in the diagnosis (Figures 4B,C). Immunohistochemistry: Tg (−), TTF-1 (−), P53 (−), CyclinD1 (partial +), CK19 (+), galectin-3 (+), HBME-1 (−), CK7 (−), P40 (+), CK (+), NapsinA (−), CK5/6 (+), P63 (+), Vim (−), CD5 (+), CD117 (+), CgA (−), Syn (−), CD56 (−), and Ki67 (about 50%+) (Figures 4E,F). HE morphology and immunohistochemistry were consistent with thymoid-differentiated carcinoma. PET-CT results showed that the right lobe of thyroid was obviously enlarged and PDG metabolism was increased, which was considered to be malignant tumor (thyroid cancer). There were several small lymph node shadows in the left and right supraclavicular fossa of the neck, with increased fluorodeoxyglucose (FDG) metabolism, and metastasis was considered. Multiple small nodules in both lungs, no abnormal FDG metabolism, combined with occupational history, silicosis was considered. Total thyroidectomy and right central cervical lymph node dissection were performed under general anesthesia. During the operation, a solid yellow-white fish nodule with a size of 5 × 4 cm was observed in the right lobe of the thyroid gland, which was hard, with poor mobility and unclear boundary. Thyroid cancer was considered, and the tumor infiltrated the trachea, esophagus, right internal jugular vein, and right common carotid artery. No nodules were found in the right lobe and isthmus of the thyroid gland. Multiple enlarged lymph nodes were seen in zones II, III, and IV of the right neck. The tumor infiltrated the trachea, esophagus, right internal jugular vein, and right common carotid artery. Radical resection could not be performed, so palliative resection was performed after communication with the family. The thyroid gland was cut off from the left side of the isthmus, and the pretracheal tissue was dissociated and pulled to the left side, to separate the upper pole of the right lobe of the thyroid gland. The superior thyroid artery and vein were ligated, and the thyroid gland was dissociated laterally. The thyroid gland was free laterally. It infiltrated the right common carotid artery, esophagus, and trachea, and could not be separated. Some tumor tissue remained. Most of the mass was removed. The left lobe of the thyroid gland was completely removed by the same method, and the left recurrent laryngeal nerve was explored. The right neck was explored and lymphatic tissue in the right II, III, and IV areas was removed. Routine postoperative pathology: (1) (Right lobe of thyroid) thymoid-differentiated carcinoma was shown. (2). (Left lobe of thyroid) no cancer was found in the submitted tissue. (3). Lymph node metastasis (right neck) (2/11). More than 4 months after surgery for thyroid cancer, he was readmitted because of “dyspnea for 1 week and aggravation for 2 days.” The reexamination of neck and chest CT showed the following: “more than 4 months after thyroid cancer surgery,” (1) the right thyroid area occupied space, combined with the history, considering the possibility of tumor recurrence and involvement of trachea and esophagus; it is recommended to combine enhanced scan; and (2) multiple nodules were present in both lungs. Compared with 2019-09-16 chest CT, nodules increased and some were enlarged, which was considered as metastatic tumor. Under emergency local anesthesia, the patient was treated with “tracheotomy and endotracheal intubation.” The patient was discharged with a metal cannula after surgery.

## 2. Discussion

Thyroid carcinoma showing thymus-like differentiation (CASTLE) is a very rare type of thyroid cancer, and only about 100 cases have been reported globally. In 1985, it was first reported by Miyauchi et al. (1). In 1991, Chan and Rosai classified tumors with thymoid differentiation in thyroid and surrounding cervical soft tissues into four types: benign ectopic hamartomatous thymoma, ectopic cervical thymoma, spindle cell tumor with thymus like differentiation (SETTLE), and CASTLE (2). CASTLE was officially classified as an independent thyroid tumor type by the WHO in 2004. At present, it is believed that CASTLE may be derived from ectopic thymus or gill sac residual tissue, which usually has an insidious onset and is mostly found in the form of neck mass, and can also be seen for treatment in the form of hoarseness and dyspnea (3, 4). Preoperative imaging and cytological diagnosis of this disease are difficult, and it is easy to be confused with thyroid squamous cell carcinoma, poorly differentiated or undifferentiated thyroid carcinoma, thyroid metastatic lymphoepitheliomatoid carcinoma, metastatic squamous cell carcinoma, and thymic carcinoma (5, 6). Most literature studies show that CASTLE has a slow course of disease and a good prognosis. According to the study by Roka et al. and Piacentini et al., for CASTLE patients without lymph node metastasis, surgery seems to be enough, because no patient relapsed in their study (7, 8). In a study conducted by Tsutsui et al., two patients without lymph node metastasis and without postoperative radiotherapy were followed up for 5 and 10 years, respectively, and no tumor recurrence was found (9). Stanciu et al. performed total thyroidectomy and neck dissection for a patient with thymoid-differentiated carcinoma, and there was no local recurrence or distant metastasis after 2.5 years of follow-up (10). However, a Chinese scholar retrospectively analyzed two patients with CASTLE. One patient underwent total thyroidectomy plus neck dissection and postoperative Intensity-modulated radiation therapy (IMRT), and died of brain metastases 13 months after surgery. In this paper, patient 2 suffered from dyspnea caused by tumor recurrence and compression of the trachea more than 4 months after the operation. The patient underwent tracheotomy to relieve the symptoms and was discharged with a metal cannula after the operation. This indicates that some patients still have rapid recurrence and metastasis, which is even life-threatening. Dang et al. reported a similar case, in which the patient relapsed 5 years after surgery and refused surgery and radiotherapy, but the condition remained stable after 15 months of follow-up after tracheotomy to relieve dyspnea caused by tracheal compression (11). Ito et al. (12) and Sun et al. (13) both found that 50%–60% of CASTLE patients were accompanied by cervical lymph node metastasis and surrounding tissue and organ invasion, and believed that the scope of surgery should at least include the affected side of the thyroid lobe plus selective cervical lymph node dissection plus surrounding affected tissues and organs. Huang et al. believed that the prognostic factors of CASTLE included lymph node metastasis, infiltration of peripheral tissues outside the gland, and postoperative radiotherapy. Ge et al. (14) analyzed 82 cases of CASTLE and concluded that lymph node metastasis was not associated with recurrence, while postoperative radiotherapy could not reduce recurrence. However, it has been reported in relevant literature that CASTLE is sensitive to radiotherapy (15), and the local recurrence rate of patients who received both surgery and postoperative radiotherapy is extremely low. In the case of Tsutsui et al. (9), one patient refused surgery and only received radiotherapy. The tumor was in complete remission, and no recurrence was found on CT scan during the 7-year follow-up. Of the 10 patients with CASTLE tumor who underwent surgery and adjuvant radiotherapy, only four recurred, all outside the irradiated area. In case 1, the metastatic lymph nodes in the lateral cervical region were reduced after radiotherapy, which proved that radiotherapy had a certain effect. The role of chemotherapy in CASTLE is still unclear (13). Hanamura et al. reported that CASTLE patients with lung metastases were significantly relieved or even disappeared after first-line chemotherapy with cisplatin and doxorubicin and second-line chemotherapy with carboplatin and paclitaxel (3). It was found that the immunohistochemical markers CD5 and CD117 associated with thymic tumors were strongly positive in CASTLE, with a positive rate close to 100%. However, Tg, TTF-1, and calcitonin were almost not expressed, suggesting that the tumor had thymic differentiation characteristics rather than thyroid differentiation, thus distinguishing it from other thyroid tumors, which was consistent with previous reports. P63 is also widely expressed in CASTLE, so the combined examination of CD5, CD117, and P63 can improve the diagnostic accuracy of CASTLE. Thomas De Montpréville et al. found that glucose transporter-1 (GLUT-1) and Pax8 are also helpful for the diagnosis of intrathyroid thymic carcinoma (ITTC); the expression of GLUT-1 in ITTC is highly specific, and Pax8 can be used for auxiliary diagnosis (16). p63 gene, Bcl-2, EGFR, p53, and other markers may be involved in the tumorigenesis and development of CASTLE and have been reported as biomarkers of CASTLE (17). Recently, it has been reported that S-100A9 is a marker of squamous cell origin and is diffusely expressed in thyroid squamous cell carcinoma and undifferentiated carcinoma with squamous cell components, while it is only scattered and focally expressed in CASTLE, which is helpful for the differentiation between them (12). Thyroid squamous cell carcinoma and undifferentiated carcinoma have a high degree of malignancy and are treated differently from thymoid-differentiated carcinoma (18, 19). The different diagnosis and treatment processes for patients will directly affect the prognosis of patients, so it is particularly important to distinguish them.

## Data Availability

The datasets presented in this study can be found in online repositories. The names of the repository/repositories and accession number(s) can be found in the article/Supplementary Material.
